# *In silico* analysis of pressure distribution and flow profiles across an experimental left ventricular assist device accessory

**DOI:** 10.1093/icvts/ivaf031

**Published:** 2025-02-25

**Authors:** Anna Osypka, Florian Meissner, Deniz Ozturk, Roxane Windisch, Heiko Vestner, Michelle Costa Galbas, Martin Czerny, Wolfgang Bothe

**Affiliations:** Department of Cardiovascular Surgery, Medical Center-University of Freiburg, Faculty of Medicine, University of Freiburg, Hugstetterstr. 55, 79106 Freiburg, Germany; Department of Cardiovascular Surgery, Medical Center-University of Freiburg, Faculty of Medicine, University of Freiburg, Hugstetterstr. 55, 79106 Freiburg, Germany; Department of Medical Simulations, Medividia, Herent, Belgium; Department of Cardiovascular Surgery, Medical Center-University of Freiburg, Faculty of Medicine, University of Freiburg, Hugstetterstr. 55, 79106 Freiburg, Germany; Department of Cardiovascular Surgery, Medical Center-University of Freiburg, Faculty of Medicine, University of Freiburg, Hugstetterstr. 55, 79106 Freiburg, Germany; Department of Cardiovascular Surgery, Medical Center-University of Freiburg, Faculty of Medicine, University of Freiburg, Hugstetterstr. 55, 79106 Freiburg, Germany; Department of Cardiovascular Surgery, Medical Center-University of Freiburg, Faculty of Medicine, University of Freiburg, Hugstetterstr. 55, 79106 Freiburg, Germany; Department of Cardiovascular Surgery, Medical Center-University of Freiburg, Faculty of Medicine, University of Freiburg, Hugstetterstr. 55, 79106 Freiburg, Germany

**Keywords:** heart failure, mechanical circulatory support, left ventricular assist device, computational fluid dynamics, medical device development

## Abstract

**OBJECTIVES:**

Implantation of left ventricular assist devices conventionally requires a sternotomy and cardiopulmonary bypass. An experimental accessory was designed to redirect the device’s outflow graft through the left ventricle into the ascending aorta. This design allows for implantation via left thoracotomy only but resulted in significant pressure loss both *in vitro* and *in vivo*. We evaluated the reasons for the pressure loss of the experimental accessory by quantifying pressure distribution and flow profiles using computational fluid dynamics simulation tools.

**METHODS:**

A computational fluid dynamics model based on the accessory’s geometry was used to simulate nominal blood flow through the model. Quantities of interest included pressure and flow velocity. Pressure differences between the pump inlet and outlet were calculated at different rotational speeds (4000, 5200, 6400 rpm) and pump flow rates (1, 5, 8.4 L/min). Results were compared with simulations of a generic left ventricular assist device to determine the accessory’s impact.

**RESULTS:**

Natural pump characteristics were observed, as increased rotational speed caused an increase in pressure head with a constant flow rate. For all cases, a greater decrease in pressure head was seen between 5 and 8.4 L/min than between 1 and 5 L/min. Curvature intensity and channel bifurcation in the outflow were the main contributors to downstream pressure loss.

**CONCLUSIONS:**

The next iteration of the left ventricular assist device accessory should focus on minimizing curvatures and avoiding bifurcations in the outflow. Further development may allow for less invasive left ventricular assist device implantation with negligible alterations in pump performance.

## Graphical abstract

**Figure:**
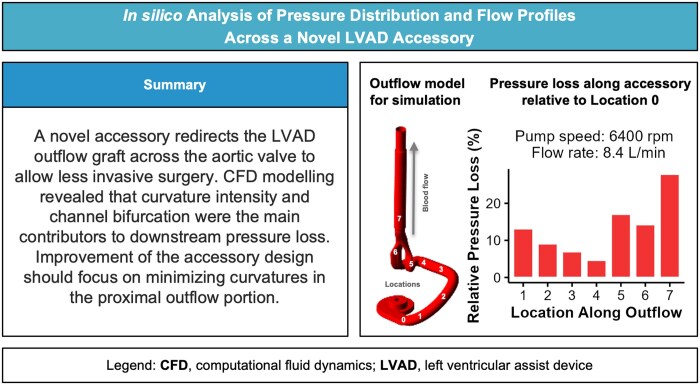

## INTRODUCTION

**Figure 1:**
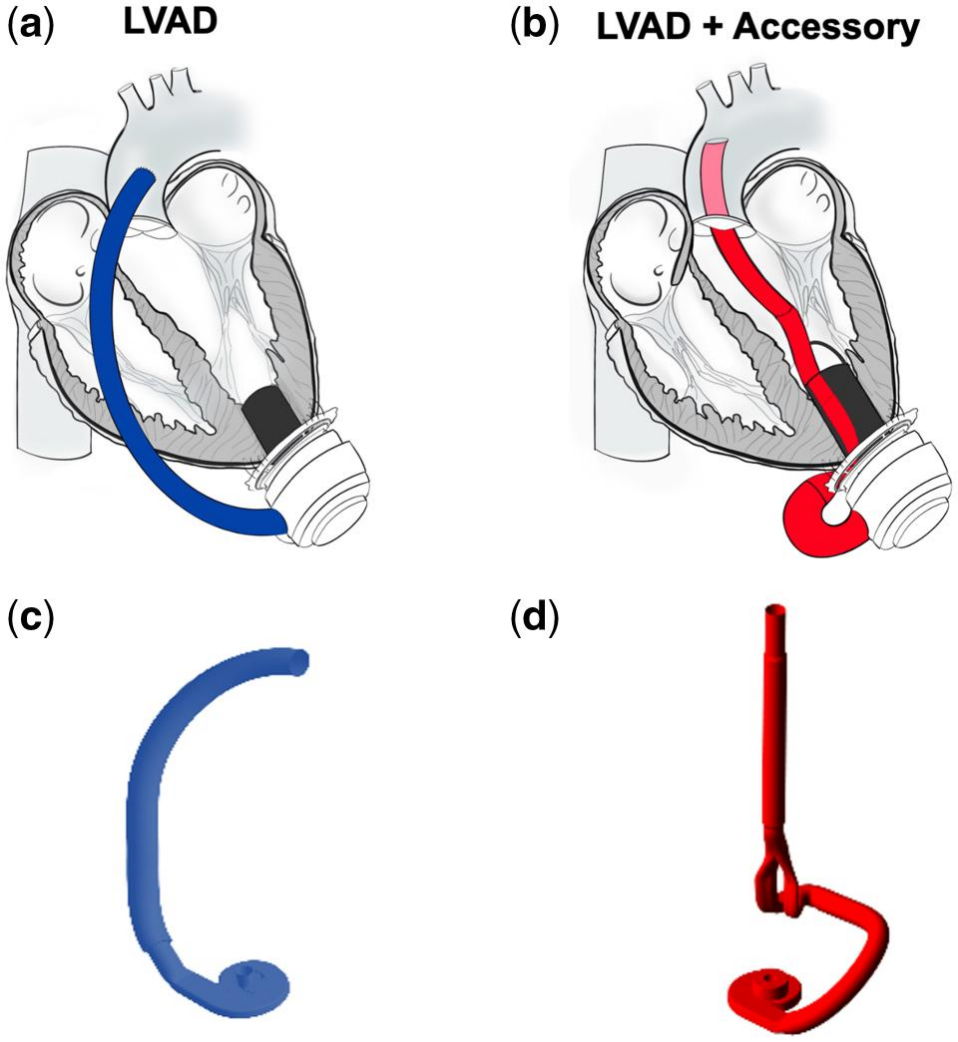
Left ventricular assist device (LVAD) schematic without (**a**) and with accessory (**b**). Images in (**a**) and (**b**) show the inflow in black, and the outflows match the volume models for the LVAD and LVAD with accessory in (**c**) and (**d**), respectively. The schematics are reprinted from ‘Impact of an Accessory for Left Ventricular Assist Devices on Device Flow and Pressure Head In Vitro’ by Meissner *et al*. [1]

Conventional left ventricular assist device (LVAD) implantation requires either a full or partial sternotomy to suture the outflow graft to the aorta (Fig. 1a). A novel, experimental blood-guiding accessory that redirects the LVAD outflow transventricularly via the left ventricle (LV) apex into the ascending aorta was designed to allow LVAD implantation solely via a left thoracotomy (Fig. 1b). The accessory was designed with computer-aided design software, and this file was then used to create a volume model (Fig. 1c and d) for *in silico* testing and a 3D-printed version printed in titanium for animal testing. The experimental LVAD accessory has a bifurcation in the proximal outflow that subsequently recombines into a single channel connected distally to a transventricular stent graft, as shown in the volume model in Fig. 1d. This bifurcated design allows for a smaller implantation diameter for the accessory than a configuration with two adjacent round channels for inflow and outflow. *In vitro* and *in vivo* testing revealed significant pressure loss for the LVAD with the accessory compared to an LVAD without the accessory [1, 2]. The reasons for this pressure loss were, however, unknown.

Computational fluid dynamic (CFD) simulations have become an important tool for simulating blood flow within mechanical circulatory support devices, including LVAD pumps. Studies using CFD assessment have, e.g. shown that pulsatile-flow support devices have several advantages, such as lower pressures in the ascending aorta and less wall shear stress than continuous flow pumps [3]. The HeartMate 3 (HM3) fluid–rotor interactions have been investigated thoroughly using CFD simulation to demonstrate that the artificial pulse of the HM3 improves the pump washout and to show the effects of pump design on flow fields, showing good agreement between CFD analysis and particle imaging flow visualization and overall clean flow fields [4, 5].

In this study, we aimed to evaluate the reasons for the pressure loss of the LVAD accessory by quantifying pressure distribution and flow profiles using CFD simulation.

## MATERIALS AND METHODS

### Model development workflow

The HM3 was the chosen generic LVAD for the simulations. Models of the HM3 (Control) and HM3 with accessory (Accessory) were created using computer-aided design software (SolidWorks, SolidWorks Corp., Vélizy-Villacoublay, France). The models were used to generate volume models for *in silico* testing. The volume model only included the areas in contact with blood, which were extracted as triangulated representations (STL format), a format easily implemented within the simulation software. The inlet and outlet openings were closed with an additional surface, as only liquid-tight volumes can be used for finite-volume CFD solvers. The model geometry was checked for mesh inaccuracies with 3DTransVidia software (Capvidia, Houston, TX). The verified geometry was imported into the CFD software FlowVision (Medividia, Herent, Belgium) for pre-processing, solving and post-processing. The flow solver, the numerical method of solving the calculations for these simulations, was an implicit/explicit all-Mach number, general-purpose Navier-Stokes solver with a segregated solution approach [6]. Further information about the general setup can be found in the supplementary material provided, including a detailed description of the final computational grid, seen in Supplementary Material, Fig. S1, and supplementary simulations done to prove the accuracy of the established approach, seen in Supplementary Material, Fig. S2.

### Boundary conditions

The flow channel only has two openings. The mass flow rate was calculated based on the volumetric flow rate. The mass flow was used as an inlet boundary condition and allowed for the specification of the exact pump flow, which cannot be done using the natural method of specifying pressures. A free outlet type of boundary condition with zero pressure was used. As there were no flexible boundaries in the domain, the use of relative pressure difference was suitable. The inner channel and impeller surfaces were considered no-slip walls for the simulations.

### Working fluid

The continuous phase of liquid blood was assigned as working fluid and modelled as a Newtonian fluid. Nominal blood properties were assumed [molar mass: 0.02 kg/mol, density: 1060 kg/m^3^, viscosity: 0.004 kg/(m × s)].

### Simulations

The HM3 was simulated at three rotational speeds (4000, 5200, 6400 rpm) and flow rates (1, 5, 8.4 L/min), without (Control) and with the LVAD accessory (Accessory). The simulations were run until a stable transient state of oscillations was reached (i.e. dynamic convergence). The simulations were run for ∼5000 steps, resulting in data for 0.2 s. All pressures were averaged using the last 0.05 s of the simulation data. The pressure head was calculated as the difference between outlet and inlet pressure. During post-processing, pressure and velocity fields and the average pressure head were calculated to compare the operating points. The evolution of the total pressure along the flow channel downstream of the impeller was tracked via numerical sensors. This was done to understand the local contribution to pressure loss.

## RESULTS

**Figure 2:**
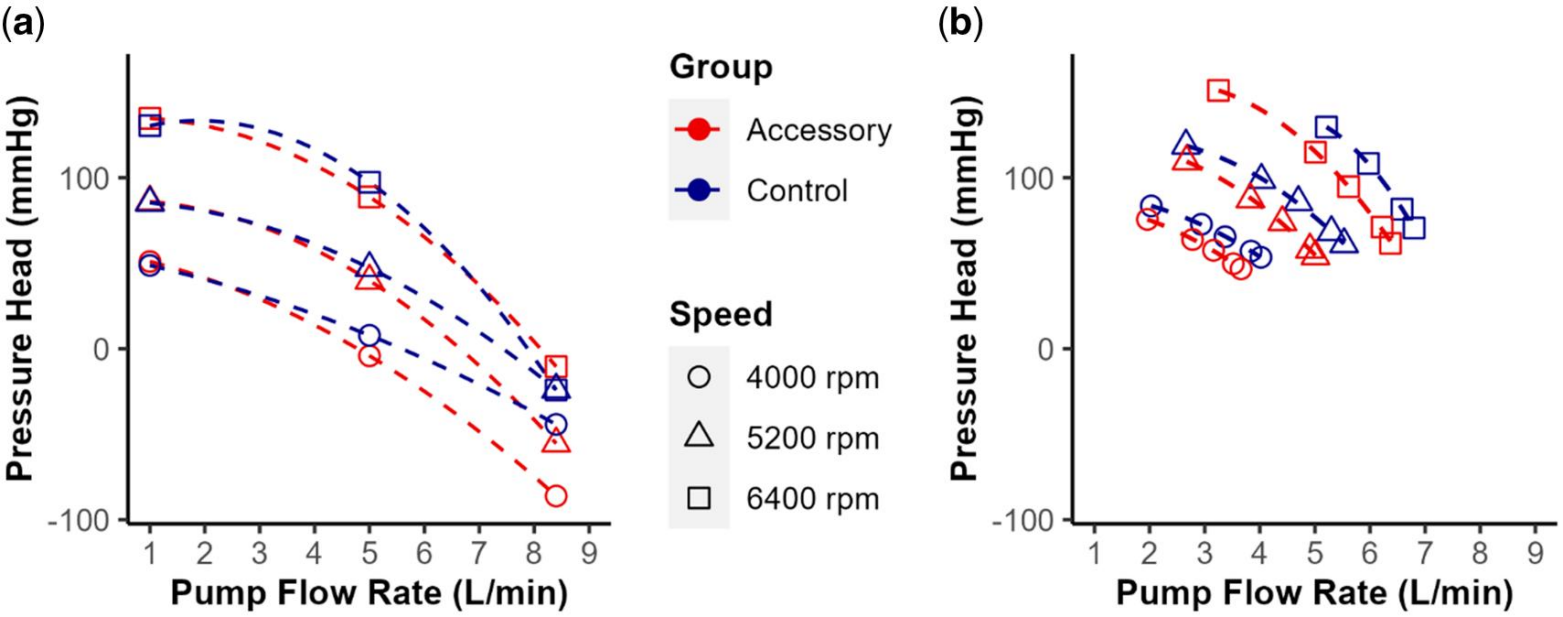
Comparison of H-Q curves from *in silico* and *in vitro* testing. (**a**) Results from computational simulation. (**b**) Previously published results from mock circulation loop testing [1]. For both (**a**) and (**b**), the shapes correspond to different speeds, and the colours represent the group tested, Control or Accessory

A natural pump response was observed for both groups as the pressure head decreased with an increased pump flow rate and vice versa. Simulation cases at 1 L/min showed similar pressure head values for the Control and Accessory (Fig. 2a). For the Accessory group, the pressure head decreased more extensively for flow rates between 5 and 8.4 L/min than between 1 and 5 L/min. Pressure head values reached negative values for flow rates at 5 and 8.4 L/min for 5200 and 6400 rpm.

**Figure 3:**
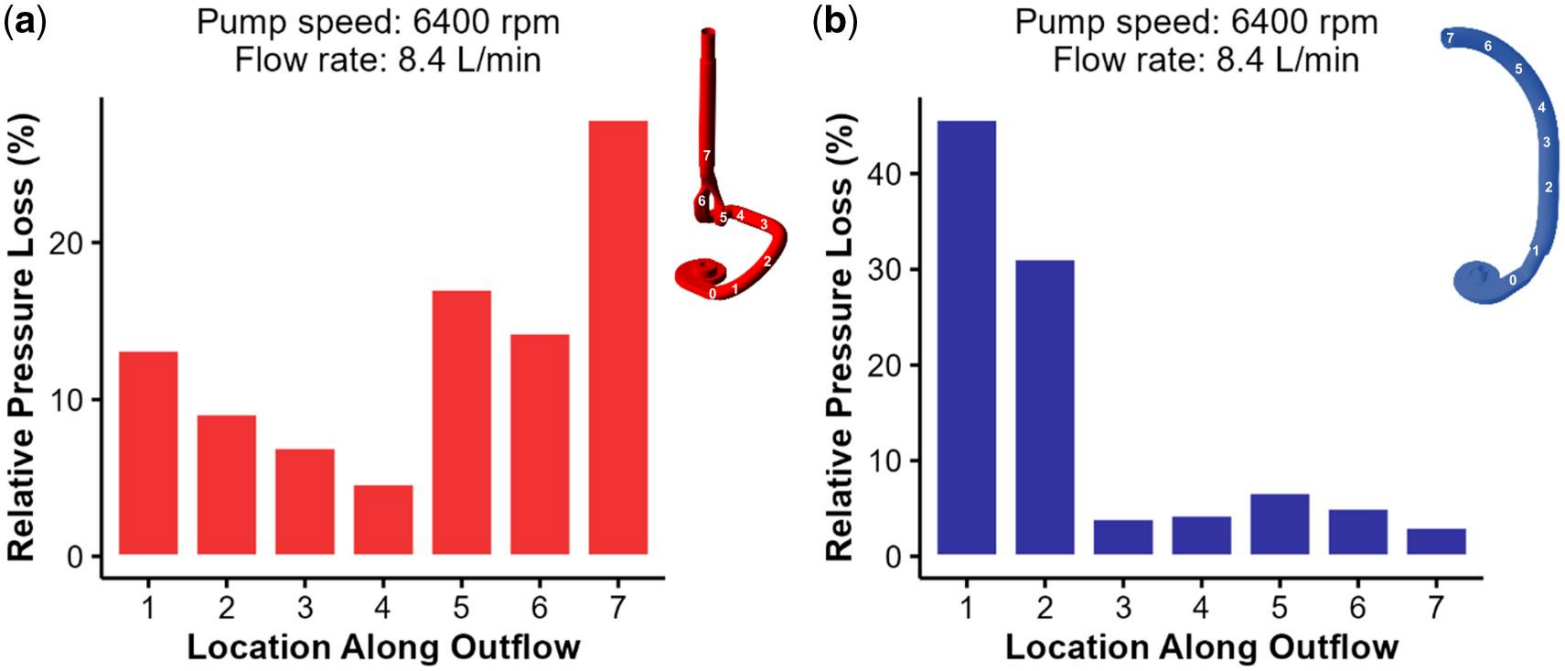
Average pressure loss in the Accessory and Control groups at a flow rate of 8.4 L/min and 6400 rpm. (**a**) Loss with accessory. (**b**) Control. The *x*-axis represents the location in the volume model seen to the right of each graph, and the numbers correspond to the locations labelled on the volume model. The *y*-axis describes the relative pressure loss within the volume model

Figure 3 shows the selected locations and associated % pressure loss regarding overall loss for a high-velocity and high-flow case. In Fig. 3a, the highest pressure loss for the accessory is observed distal to the channel bifurcation and recombination, which were characterized by a significant change in curvature and diameter. The pressure loss was most significant at the proximal HM3 impeller outlet, with minimal loss in the distal outflow, as seen in Fig. 3b.

**Figure 4:**
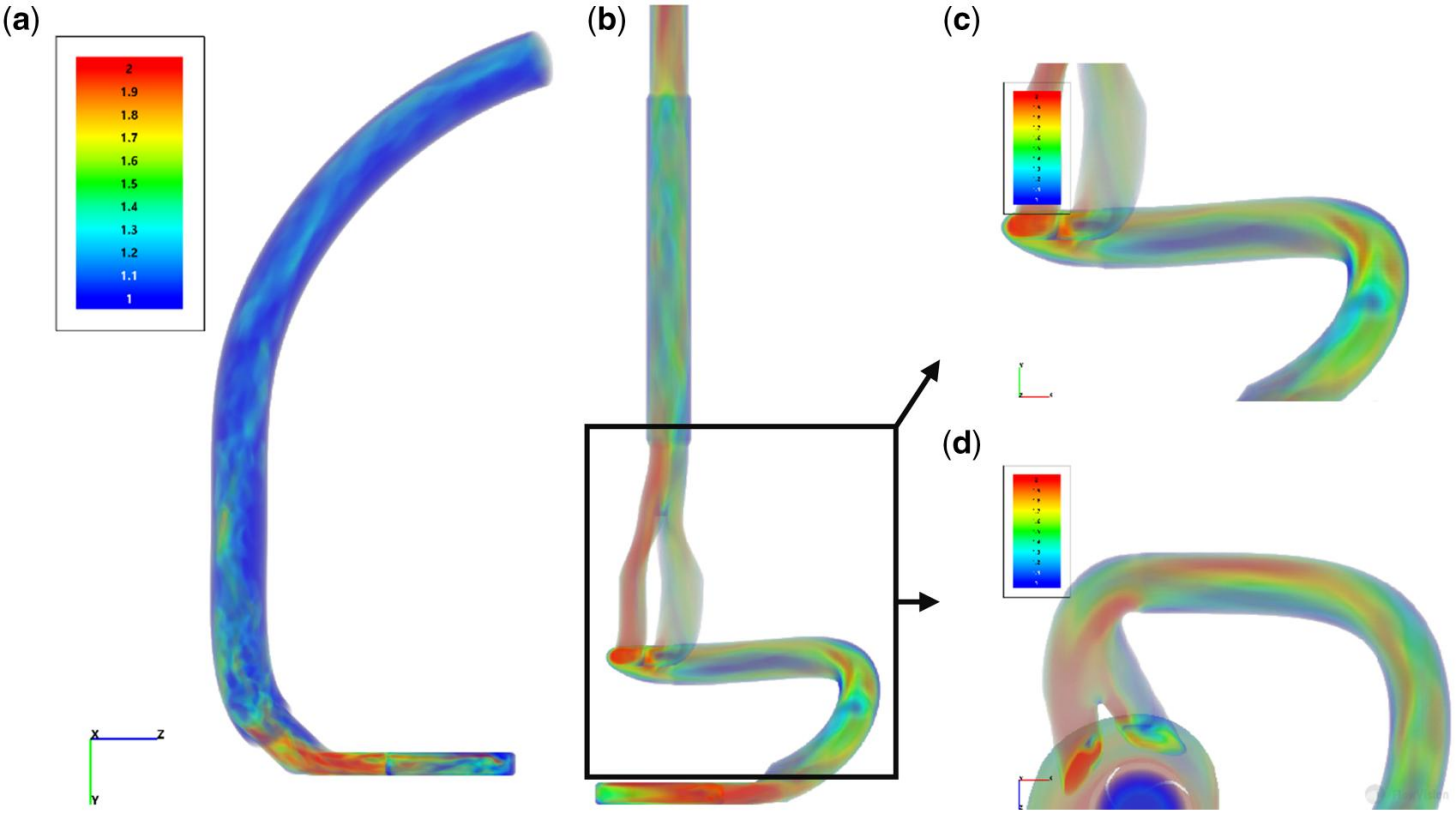
Instantaneous velocity captured between two blade passages at 6400 rpm and 8.4 L/min. (**a**) Accessory group. (**b**) Control. (**c**) Closer look at the accessory outflow. (**d**) Channel bifurcation and severe curvatures. (**e**) Distal outflow of the Control with the highest velocity and uneven velocity distribution

Visualizations of the instantaneous flow field (velocity and total pressure) for an operating point at 6400 rpm and 8.4 L/min, with corresponding impeller revolution periods, are given in Fig. 4 for both the Control and the Accessory. The velocity subfigures show that the highest velocity reached within each volume model is within the HM3 pump, as seen in the overview images (Figs 3b and 4a). Comparatively, the accessory shows additional areas of high velocity that were reached around the channel bifurcation and the areas of sharp curvature (see Fig. 4c and d). The flow rates within the accessory’s channel bifurcation were higher in the outer channel of the bifurcation (see Fig. 4d) due to the rotation relative to the flow direction, as expected. The HM3 outflow showed a relatively steady and well-distributed velocity field throughout the channel.

## DISCUSSION

The principal findings of this *in silico* study showed that the novel accessory caused additional pressure loss. The use of CFD simulations showed that the sharp curvature of the accessory outflow design and the bifurcation in the model were the main contributors to pressure loss. The pressure head reached negative values for the Accessory and Control cases, specifically at flow rates higher than 5 L/min and lower rotational speeds. As the pressure head reached values near zero, the pump reached its operational maximum flow rate. The negative values in the results show that the pump operation is only feasible if another source contributes power to the system. It is essential to consider the residual function of the LV when adjusting to high flow rates, as these high flow rates often need the compensational function of the LV to maintain proper function. The residual function could be impaired due to the implantation of the accessory. It should be considered a point of interest for further simulations, including simulations of a functional LV for designs that show the most significant potential. Apart from this, natural pump response was seen as the pressure head decreased with increased flow rate. Natural pump response refers to this change in pressure head with respect to flow for centrifugal pumps [7].

Moreover, there was a significant difference in flow velocities in the channels created by the bifurcation. A study by Valsala *et al.* [8] evaluated how curvatures affect pressure and velocity contours within a pipe and assessed the possibility of reducing pressure loss by including guide vanes within the pipe curvatures. This would pose a possible solution if the accessory design needs intense curvature due to the limitation of space and heart dimensions. The insertion of vanes within the curvatures could be studied and implemented as a solution to reducing pressure loss, given that the manufacturing challenges are adequately addressed. To minimize pressure losses, curvatures should be kept to a minimum, and the design should focus on smooth curvatures, if necessary, rather than abrupt, elbow-like turns. However, incorporating these changes may impact the accessory size and the implantation method; thus, future design improvements should simultaneously consider all aspects, limitations and challenges.

The simulations allowed for a closer analysis of the experimental LVAD accessory characteristics and a comparison to the base LVAD at varying rotational speeds and flow rates, allowing for a well-established baseline of the simulation setup, methods and execution. Thamsen *et al.* included a much more refined mesh size, simulating the HM3, but a more limited range of flow rates simulated. The visualization of the velocity magnitude within the HM3 model showed similar flow properties to the current study, with relevant stagnation in curvature regions [9].

Previously, the impact of the accessory on LVAD performance was evaluated *in vitro* in a mock circulatory loop. The pressure head and flow rate were lower than for the Control, as seen in the *in silico* assessment of higher flow rates. The *in vitro* data did not show as much of an increase in pressure head difference between both groups as the flow rate was increased [1]. *In vivo* testing revealed no significant thrombus formation and no relevant aortic valve dysfunction in the acute phase of implantation [2].

The current study focused on the integral performance characteristics of the accessory and control design (HM3). In the future, thrombogenic footprint and blood damage can be studied via *in silico* methods; however, this would require separate numerical verification (e.g. mesh convergence) to focus on the solution of additional variables such as wall shear stress and blood residence time. Wiegmann *et al.* [10] analysed the effect of an artificial and residual cardiac pulse on flow in the HM3. For the current study, the data gathered resembled only the initial cases seen in the work of Wiegmann *et al.* For future simulations, LV function and residual wall motion will play a key role in the realistic evaluation of LV function through simulations. It would be of great interest to investigate other important characteristics, such as thrombogenicity and haemolysis, once an accessory iteration is deemed well-suited after preliminary tests. Boraschi *et al.* studied the thrombogenic profile of different contemporary centrifugal-flow pumps, such as the HeartWare Ventricular Assist Device (Medtronic, Minneapolis, MN) with its Lavare Cycle and the HM3 with its artificial pulse. It was shown how speed modulation affects washout areas, stagnation and shear stress [11]. For future simulations, gaining insight into these variables through CFD simulation could allow for a more in-depth assessment of the accessory iteration in combination with different LVAD systems, including the EVAHEART 2 (EvaHeart, Bellaire, TX), which might be approved for clinical use by the Food and Drug Administration (FDA) in the near future [12]. The EVAHEART 2 has been assessed *in silico* before by May-Newman *et al.* [13]. As the use of CFD and other technical expertise, such as artificial intelligence, continues to gain relevance, it is important to understand the medical side of this innovative LVAD accessory and the technical aspects of testing it.

### Limitations

For this study, the computational cost was an important factor in choosing the amount of simulation time allotted for each simulation. Generally, the longer a simulation was run, the higher the chances of convergence to a stable simulation. As a compromise between both, we decided on a grid with a relative error of 1.2%. If haemolysis and thrombus formation are to be studied, higher spatial and temporal discretization levels would be needed, increasing the time needed per simulation.

Only a limited number of variables were tested in the anticipated areas of pressure loss. Although our results align with the natural pump response, further simulations should cover more variables of interest and assess the areas of indispensable curvature in more depth.

## CONCLUSION

CFD simulation allowed a preliminary characterization of the blood flow of an experimental LVAD accessory. It demonstrated that curvature intensity and channel bifurcation in the outflow tract were the main contributors to downstream pressure loss. Further design optimizations should focus on minimizing curvatures in the outflow portion of the design. *In silico* approaches represent a cost-effective way to virtually test multiple design iterations before prototyping and physical testing.

## Supplementary Material

ivaf031_Supplementary_Data

## Data Availability

The data underlying this article will be shared on reasonable request to the corresponding author.

## Notes

Presented at the 37th EACTS Annual Meeting, Austria, Vienna, October 6, 2023.

## ABBREVIATIONS

CFD: Computational fluid dynamics
FDA: Food and Drug Administration
HM3: HeartMate 3
LV: Left ventricle
LVAD: Left ventricular assist device

## References

[1] Meissner F , Eichelkraut D, Schimmel M et al Impact of an accessory for left ventricular assist devices on device flow and pressure head in vitro. Bioengineering 2023;10:486. 37106673 10.3390/bioengineering10040486 PMC10135582

[2] Meissner F , Galbas MC, Straky H et al In vivo testing of a second-generation prototype accessory for single transapical left ventricular assist device implantation. Bioengineering 2024;11:848. 39199805 10.3390/bioengineering11080848 PMC11351186

[3] Karmonik C , Partovi S, Schmack B et al Comparison of hemodynamics in the ascending aorta between pulsatile and continuous flow left ventricular assist devices using computational fluid dynamics based on computed tomography images. Artif Organs 2014;38:142–8. 23889366 10.1111/aor.12132

[4] Burgreen GW , Loree HM, Bourque K et al Computational fluid dynamics analysis of a maglev centrifugal left ventricular assist device. Artif Organs 2004;28:874–80. 15384992 10.1111/j.1525-1594.2004.07384.x

[5] Fang P , Du J, Boraschi A et al Insights into the low rate of in-pump thrombosis with the HeartMate 3: does the artificial pulse improve washout? Front Cardiovasc Med 2022;9:775780. 35360020 10.3389/fcvm.2022.775780 PMC8962620

[6] Ozturk U , Soganci S, Akimov V, Tutkun O, Aksenov A. Validation of FlowVision CFD on ICCS2015 test case: application of gap model and SGGR for leakage flow prediction in a dry screw compressor. IOP Conf Ser: Mater Sci Eng 2019;604:012010.

[7] Princer K , Pump design and mechanics. In: Stewart S, Blood P (eds). A Guide to Mechanical Circulatory Support [Internet]. Cham: Springer International Publishing, 2022, 13–28. https://link.springer.com/10.1007/978-3-031-05713-7_2 (27 June 2024, date last accessed).

[8] Valsala RR , Son SW, Suryan A, Kim HD. Study on reduction in pressure losses in pipe bends using guide vanes. J Vis 2019;22:795–807.

[9] Thamsen B , Gülan U, Wiegmann L et al Assessment of the flow field in the HeartMate 3 using three-dimensional particle tracking velocimetry and comparison to computational fluid dynamics. ASAIO J 2020;66:173–82. 30883404 10.1097/MAT.0000000000000987

[10] Wiegmann L , Thamsen B, de Zélicourt D et al Fluid dynamics in the HeartMate 3: influence of the artificial pulse feature and residual cardiac pulsation. Artif Organs 2019;43:363–76. 30129977 10.1111/aor.13346

[11] Boraschi A , Bozzi S, Thamsen B et al Thrombotic risk of rotor speed modulation regimes of contemporary centrifugal continuous-flow left ventricular assist devices. ASAIO J 2021;67:737–45. 33074865 10.1097/MAT.0000000000001297

[12] Slaughter M , Meyer D, Ravichandran A et al The COMPETENCE trial: prospective multi-center randomized study for evaluating the EVAHEART 2 left ventricular assist system. J Heart Lung Transplant 2022;41:S25.

[13] May-Newman K , Montes R, Campos J et al Reducing regional flow stasis and improving intraventricular hemodynamics with a tipless inflow cannula design: an *in vitro* flow visualization study using the EVAHEART LVAD. Artif Organs 2019;43:834–48. 31038753 10.1111/aor.13477

