# Supplementary figures and images for: In silico analysis of pressure distribution and flow profiles across an experimental left ventricular assist device accessory
Source: Interdiscip Cardiovasc Thorac Surg. 2025 Feb 25;40(3):ivaf031. doi: 10.1093/icvts/ivaf031 (PMC11882303; doi:10.1093/icvts/ivaf031)

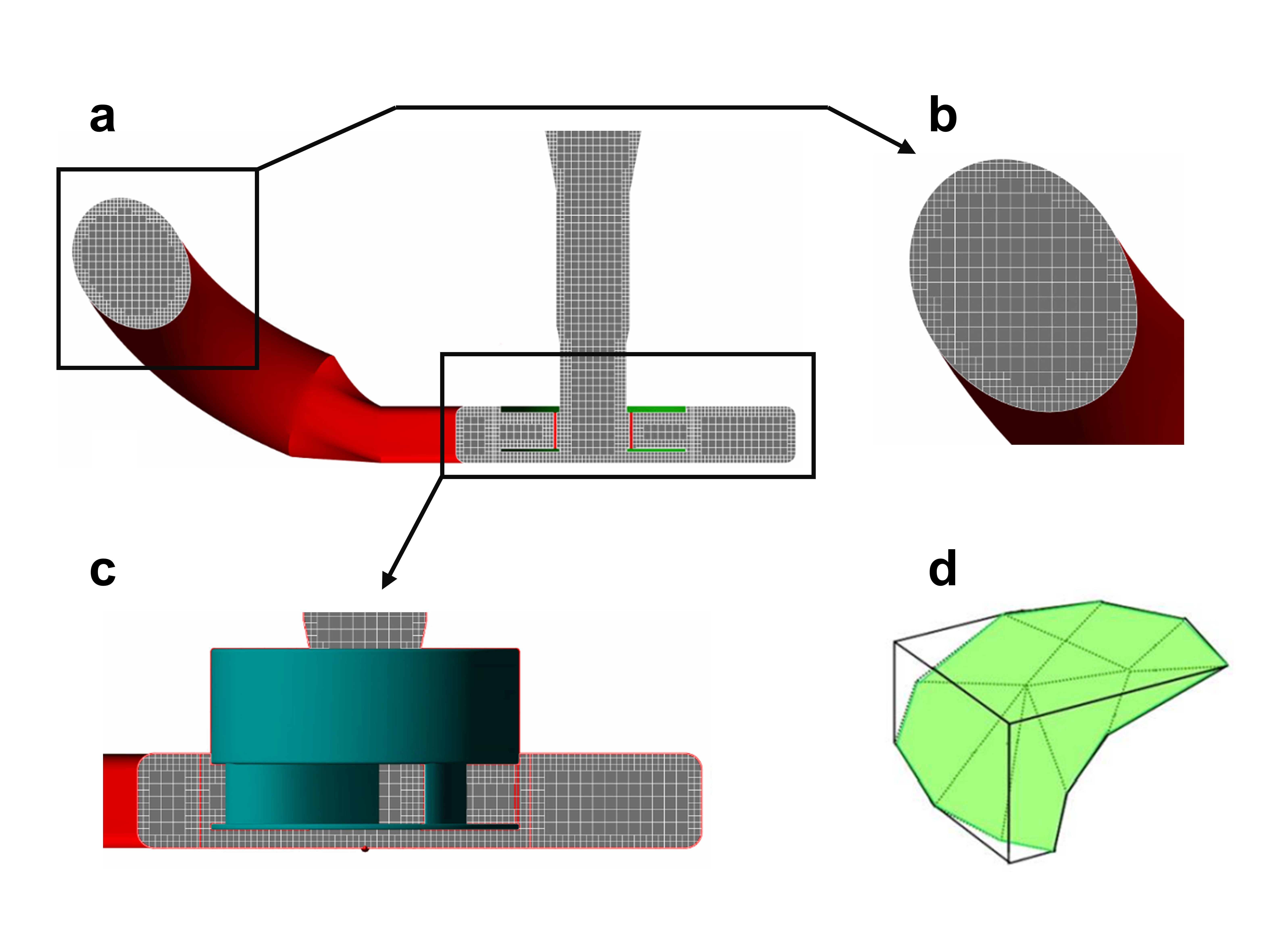

Supplement: ivaf031_Supplementary_Data [file ivaf031_supplementary_data.zip › Fig_S1.tif]

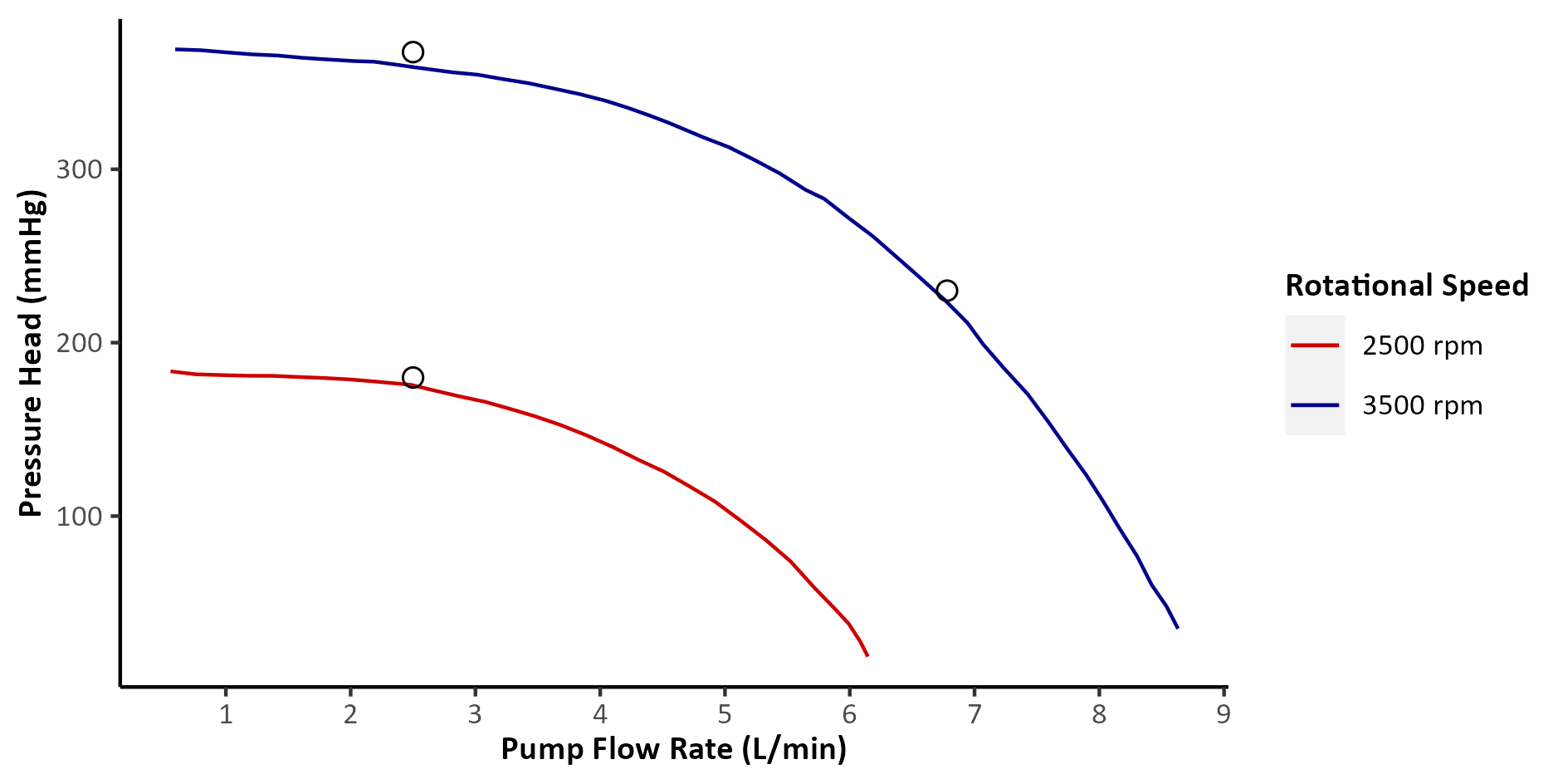

Supplement: ivaf031_Supplementary_Data [file ivaf031_supplementary_data.zip › Fig_S2.tiff]
