# Supplementary material for: In silico analysis of pressure distribution and flow profiles across an experimental left ventricular assist device accessory
Source: Interdiscip Cardiovasc Thorac Surg. 2025 Feb 25;40(3):ivaf031. doi: 10.1093/icvts/ivaf031 (PMC11882303; doi:10.1093/icvts/ivaf031)
Supplement: ivaf031_Supplementary_Data [file ivaf031_supplementary_data.zip › ivaf031_Supplementary_Data.zip]

# **SUPPLEMENT: MATERIALS AND METHODS**

### *Special discretization: computational grid*

A Cartesian grid with a sub-grid geometry resolution was used, allowing for automated grid generation. [1] From the user’s perspective, a base mesh, or initial grid, was set, and later, local surface or volume adaptations were applied to build the computational grid. The final grid for each step was built automatically, considering the impeller movement. The final grid is exemplified in Figure S1: Figure S1a shows a cut section, giving an overview of the grid and the differences in grid density throughout the volume mode. The grid density was much higher closer to the walls (Figure S1b) and the areas close to the rotor (Figure S1c), highlighting areas of greater relevance with respect to pressure loss and giving more precise calculations for these areas. The grid generation method used in this work allows for the resolution of the CAD boundaries independently from the grid resolution, allowing for automatic grid generation (Figure S1d). The initial grid was constructed with adaptations applied independently to the impeller surface and inflow and outflow channels.

**Figure S1.**

Several grid options with different overall and near-wall grid densities were studied to understand the grid convergence characteristics of the model. The initial grid picked was constructed with 120 × 70 × 90 divisions in x, y, and z directions, resulting in optimal cubic cells throughout the domain. Various adaptation levels were chosen for different surfaces. A total of three layers of adaptation were added. Levels 0, 1, and 2 counted 111, 409, and 1700 kcells, revealing a grid-related error of 12.9, 1.2, and 0.05%, respectively. Thus, the level 1 configuration was chosen as the deviations expected between different accessory designs were expected to be higher than 1%. In this case, a deviation of 1.2% was applicable for the following simulations. This level of adaptation allowed for a compromise between computational cost and acceptable variation in resulting data.

### *Numerical setup*

The rotation was modeled via a sliding surfaces approach, where the domain was divided into two or more regions and relative rotational velocity was defined. Pressure gradient calculations were performed with velocity consideration. Exact total pressure calculation was enforced in the solver instead of analytical estimation.

Turbulence was approximated with a modified k-epsilon turbulence model, and near-wall solutions were approximated with solver-specific wall functions. [2]

### *Temporal discretization: time-stepping*

Courant Friedrichs-Lewy (CFL) time stepping was used. Sliding CFL number equal to 1 was enforced throughout the simulation. Convective CFL numbers of 1, 25, 40, and 100 were tested. Accordingly, no practical difference was observed up to convective CFL = 40, which was picked for the final calculations. In addition to this, the maximum time step was limited to 1e^-4^ during the simulations to avoid any local peaks.

### *U.S. Food and Drug Administration (FDA) pump analysis*

To gain reassurance that the chosen approach for simulations was a valid option, a centrifugal-flow pump made available by the FDA was tested under the setup conditions used for this work.

Three simulated operating points were compared with the experimental data, showing reasonable agreement with a maximum relative error of 2.5% for the pressure head, as seen in Figure S2. [3,4] The grid convergence test and comparing the evaluated data points for the accessory and FDA pump reassured us to accept the data as valid. A direct comparison of the statistical significance between the control and the accessory was not applicable as the designs had two different outflows. This allowed the decision to go forward with simulations using our established approach. [3,4]

Figure S2.

# **FIGURE LEGENDS**

**Figure S1.** Example of computational grid appearance and boundary cell representation. a) Section view seen from the pump. b) Outflow channel. c) Impeller region close-up. d) Boundary cell representation. The schematic is reprinted from ‘Validation of FlowVision CFD on ICCS2015 Test Case: Application of Gap Model and SGGR for Leakage Flow Prediction in a Dry Screw Compressor.’ by Ozturk *et. al [1]*

**Figure S2.** Proposed modeling approach applied to FDA benchmark blood pump at three operating points.

# **REFERENCES**

1.	Ozturk U, Soganci S, Akimov V, Tutkun O, Aksenov A. Validation of FlowVision CFD on ICCS2015 Test Case: Application of Gap Model ^T^ M and SGGR ^T^ M for Leakage Flow Prediction in a Dry Screw Compressor. IOP Conf Ser Mater Sci Eng. 2019 Aug 1;604(1):012010.

2.	Medividia. FlowVision 3.13.02. User Guide.

3.	Ponnaluri SV, Hariharan P, Herbertson LH, Manning KB, Malinauskas RA, Craven BA. Results of the Interlaboratory Computational Fluid Dynamics Study of the FDA Benchmark Blood Pump. Ann Biomed Eng. 2023 Jan;51(1):253–69.

4.	Regulatory Catalog. Benchmark dataset for validating computational fluid dynamic (CFD) simulation of blood flow through generalized medical device geometries. 2022 Jun 9 [cited 2024 Mar 7]; Available from: https://zenodo.org/record/6629049
